# Fighting social isolation in times of pandemic COVID-19: the role of video calls for older hospitalized patients

**DOI:** 10.1007/s40520-022-02188-8

**Published:** 2022-07-06

**Authors:** Anne-Véronique Dürst, Christophe E. Graf, Carmelinda Ruggiero, Dina Zekry, Virginia Boccardi, Lauretta Monney, Isaline Joss, Karine Vuilloud, Giulia Vespignani, Wanda Bosshard, Patrizia Mecocci, Christophe J. Bula, Patrizia D’Amelio

**Affiliations:** 1 Service of Geriatric Medicine and Geriatric Rehabilitation, Department of Medicine, University of Lausanne Hospital (CHUV), Mont-Paisible 16, 1011 Lausanne, Switzerland; 2 Service of Geriatrics and Rehabilitation, Department of Rehabilitation and Geriatrics, Geneva University Hospitals, Geneva, Switzerland; 3 Geriatric and Orthogeriatric Units, Department of Medicine and Surgery, University Hospital of Perugia, University of Perugia, Perugia, Italy; 4 Division of Internal Medicine for the Aged, Department of Rehabilitation and Geriatrics, Geneva University Hospitals, Geneva, Switzerland; 5 Division of Clinical Geriatrics, NVS Department, Karolinska Institutet, Stockholm, Sweden

**Keywords:** Aging, Hospital, Communication, Social isolation, Technology, COVID 19

## Abstract

**Background:**

Loneliness and social isolation are associated with anxiety and psychological discomfort, especially amongst the oldest and fragile persons.

**Aims:**

SILVER evaluates the acceptance of video calls by old hospitalized patients and their relatives during the ban on visits due to the COVID-19. Moreover, SILVER evaluates if the use of different communication technology is associated with different outcomes in terms of anxiety, fear of self and of others’ death and mood.

**Methods:**

SILVER is an observational multicentre study. Patients hospitalized in two geriatric units in Switzerland and in one orthogeriatric unit in Italy and their relatives were enrolled. Participants can freely choose to use phone or video calls and were evaluated over a week. We measured anxiety, fear of death and mood at baseline and at the end of the study with standard scales. The use of video or phone calls was associated to a change in these parameters by two-way ANOVA for repeated measures.

**Results:**

Sixty-four patients and relatives were enrolled, 26.5% used phone calls and 73.5% video calls. The use of video calls was associated with a reduction in anxiety and fear of death in patients and relatives as compared to participants using phone calls.

**Discussion:**

Old patients and their relatives accepted and appreciated the use of video calls during hospitalization; moreover, participant using video calls appears to be less anxious and less afraid of death.

**Conclusions:**

Video calls may be a useful communication tool for hospitalized older patients to keep social relationships with relatives and reduce their anxiety and fear of death.

**Trial Registration:**

Retrospectively registered on 1st September 2021 in ClinicalTrials.gov (NCT05000099).

**Supplementary Information:**

The online version contains supplementary material available at 10.1007/s40520-022-02188-8.

## Background

Quarantine or physical isolation are used for centuries to control and limit the spread of contagious diseases [1]. Nowadays, quarantine measures have been imposed to limit COVID-19 spread. Among these measures, the ban on visits to hospitalized and institutionalized patients especially affected the most fragile older patients and has highlighted the need to prevent loneliness and social isolation[2, 3]. Indeed, the lack of social contact may significantly affect older persons’ physical [4, 5] and mental health [6, 7]. For a long, social support has been recognized as a key determinant of health [4, 5, 7, 8]. In contrast, loneliness and social isolation have been associated with negative health impacts [9], including increased anxiety and psychological discomfort [2].

During the COVID-19 pandemic, the use of social technologies and, in particular, of video calls have been proposed to nursing home residents [10, 11] and to hospitalized patients [12]. However, the efficacy of these interventions on specific patients’ outcomes [13] and the acceptance of this type of communication by older subjects [12] have still to be clarified. In particular, it remains unclear whether video calls are more effective in reducing depressive symptoms, anxiety, and fear of death among older subjects [13, 14]. Moreover, concerns have been raised about the ability and acceptance of older patients to communicate by video calls [12]. Finally, no study has been performed among older patients hospitalized in acute and post-acute care settings.

## Aims

The SILVER study aimed to evaluate the acceptance of video calls in hospitalized older patients and in their relatives during the ban on visits due to the COVID-19 pandemic. In addition, we compared patients' baseline levels and changes in anxiety, in fear of death of self, in fear of others' death, and in mood across groups choosing a different type of communication technology. Finally, we evaluated the levels of anxiety and fear of death of their loved one in relatives according to the different types of communication technology chosen.

## Methods

SILVER is an observational multicentre study. Patients were hospitalized in two acute and post-acute geriatric units in French-speaking Switzerland (University of Lausanne Medical Center in Lausanne and University of Geneva Hospital in Geneva), and in one orthogeriatric unit in Italy (Santa Misericordia Hospital in Perugia) and their relatives were enrolled in the study from 2 December 2020 to 19 May 2021.

*Study population* All patients aged 65 years and older hospitalized in the participating units over the period of visit ban due to the COVID-19 pandemic were invited to participate. Patients who agreed to participate were asked to identify a specific relative who had then agreed to participate.

*Exclusion criteria* Patients or relatives who refused to participate, and patients diagnosed with major neurocognitive disorders and a Clinical Dementia Rating [15] score of two or more were excluded.

Primary endpoints of SILVER were:

- To evaluate the acceptance of video calls as communication technology by patients and relatives.
- To compare the levels of patients’ anxiety; fear of self-death; fear of others’ death; mood according to the type of communication technique used.

Secondary endpoints of SILVER were to compare relatives’ anxiety; and fear of others’ death according to the type of communication technique used. All end points were evaluated twice: at baseline and after a 1-week period of study. *Standardization of communication:* Patients and relatives were asked to select their preferred communication support (video or phone calls). Patients who selected video calls were provided with a tablet available in each unit whereas their relatives were asked to use their own device for the calls. Video calls were performed using Skype©, WhatsApp© video, or Face-time©, according to subjects’ preferences. The process of video calls was standardized across study centers according to the position paper COMUNICOViD [16]. At least two video calls of a maximum of 15’ were organized over a single week considered as the study period. Patients were allowed to perform additional video calls alone or with the staff’s help over the study period and thereafter. Phone calls were allowed without any restriction in both groups over the study period.

### Geriatric evaluation

Each patient benefited from a comprehensive geriatric assessment at baseline; the following data were recorded: performance in basic activities of daily living (ADL) [17], in instrumental activities of daily living (IADL) [18], Mini-Mental State Examination (MMSE) [19] and Cumulative Illness Rating Scale (CIRS) [20].

### Patient’s outcomes

To evaluate their acceptance of video calls, patients were asked to rate on a 4-point Likert scale their agreement (from 1 strongly disagree to 5 strongly agree) on the following four dimensions: perceived utility; improvement in the feeling of loneliness; improvement in anxiety level; improvement in their fear of death. Total scores ranged from 4 to 20 with higher scores indicating stronger acceptance of video calls as a communication technique.

Patients’ general anxiety was assessed using the Geriatric Anxiety Scale, a 10-item form (GAS-10). The GAS-10 is a self-reported measure used to assess and quantify symptoms of anxiety in older persons; it includes 10 questions about feelings of anxiety and symptoms related to anxiety with yes/no answers. Scores range from 0 to 10, with higher scores indicating higher anxiety. A cut-off score of 6 or more defines the presence of clinically significant anxiety [21].

Anxiety related to the fear of death was evaluated by the Collett–Lester Fear of Death Scale (CL-FODS) revised form [22]. The CL-FODS assesses attitudes toward death in terms of fear of death and fear of dying (i.e., fear of the process leading to death). It considers separately self and other people’s death as well as dying and thus investigate four dimensions: Fear of Death of Self; Fear of Dying of Self; Fear of Death of Others; Fear of Dying of Others. Each dimension includes 7 items where participants are asked to rate, on a 5-point Likert format, how disturbed or distressed they are by some aspects of death and of the dying process. Answers are rated from 1 (not at all) to 5 (very). Total score for each dimension ranges from 7 to 35, with higher scores indicating higher anxiety of death and of the dying process for self- and for others’ death, respectively [22]. Results for the CL-FODS are shown as fear of self-death (composite score derived from Fear of Death of Self + Fear of Dying of Self) and fear of others (composite score derived from Fear of Death of others + Fear of Dying of others).

The 5-item Geriatric Depression Scale (GDS-5) was used to assess patients’ mood. The GDS-5 comprehends 5 questions that evaluate depressive symptoms with yes/no answers [23]. Total score ranges from 0 to 5, with higher scores indicating more depressive symptoms and a cut-off of 2 or more suggests clinical depression [23, 24].

### Relatives’ outcomes

Relatives’ acceptance of video calls was assessed using the 4-point Likert scale, previously described for patients.

The Clinical Anxiety Scale (CAS), a 25-item questionnaire about anxiety symptoms, was used to assess relatives’ anxiety. Possible scores range from 0 to 100, with higher scores indicating greater anxiety. A cut-off score of 30 or more defines the presence of abnormal anxiety [25]. As for patients, relatives’ anxiety specifically related to the fear of death and fear of dying was evaluated, by the CL-FODS revised form [22] related to others’ (namely the hospitalized loved one) death and dying.

All patient’s and relative’s outcome measures were assessed at baseline and at the end of the study period (7 days).

### Statistical analyses

No previous study compared the efficacy of video vs phone calls on patients’ anxiety, mood, and fear of death, precluding to performing a power calculation based on existing data. Hence, a post-hoc power analysis was conducted at the end of data collection on the primary and secondary endpoints by the G*power analyses software [26] to provide some insight into the study’s actual statistical power for each endpoint.

Baseline characteristics of patients who selected video vs phone calls were compared by one-way ANOVA for continuous Gaussian variables, the U Mann–Whitney test for continuous non-Gaussian variables and Fisher exact test for gender and χ^2^ test for country of enrolment.

The effects of video calls as compared to phone calls were evaluated by two-way ANOVA for repeated measures. SPSS 25.0 was used for the statistical analyses and *p* < 0.05 was considered statistically significant. Graphs were drawn using GraphPad 8.0 for Windows.

## Results

Amongst the 91 patients and relatives asked to participate in the study, 20 patients (21.9%) and 7 relatives (7.7%) refused, leaving a final sample of 64 enrolled patients, 27 recruited in Italy and 37 in Switzerland (8 in Geneva and 29 in Lausanne). No significant difference was observed between patients who refused and those who agreed to participate in mean age (84 ± 3 vs 85 ± 7, *p* = 0.371), gender (80% vs 88%, *p* = 0.361), as well as across countries (45% in Switzerland and 55% in Italy, *p* = 0.270). General characteristics of the enrolled population are reported in Table 1.

**Table 1:** Characteristics of patients according to the type of communication technology chosen

	Total study population (*N* = 64)	Type of communication technology chosen		*p* value
		Phone calls (*N* = 17)	Video calls (*N* = 47)	
Age (years)	85 ± 7	86 ± 7	84 ± 7	0.281
Education (years)	9.4 ± 4.2	8.8 ± 3.9	9.7 ± 4.3	0.442
Gender (%)	88% (F), 22% (M)	94% (F), 6% (M)	72% (F), 28% (M)	0.089
Country (%)	58% (S), 42% (I)	53% (S), 47% (I)	59 (S), 41% (I)	0.423
MMSE	22.6 ± 4.9	23.3 ± 5.7	22.3 ± 4.6	0.500
CIRS	15 ± 7	13.8 ± 7.4	15.4 ± 7.2	0.452
ADL*	5 (4–6)	4 (3–6)	3 (4–6)	0.675
IADL*	7 (1–6)	5 (1–6)	3 (1–6)	0.672
GAS-10	2.5 ± 07	**1.4 ± 1.3**	**2.4 ± 2**	**0.046**
CL-FODS fear of self-death	47.2 ± 12.8	50.3 ± 14.0	46.05 ± 12.3	0.256
CL-FODS fear of others death	68.1 ± 9.3	65.4 ± 8.2	69.1 ± 9.5	0.156
GDS-5	2.5 ± 0.7	2.4 ± 0.7	2.5 ± 0.7	0.452

Significant *p* values are in bold

Mean and standard deviations are shown for Gaussian variables, median and percentiles (25–75) are shown for non-Gaussian variables (indicated by *), non-continuous variables are shown in percentage. *p* values were calculated by one-way ANOVA for continuous Gaussian variables, by U Mann–Whitney test for continuous non-Gaussian variables and by Fisher exact test and χ^2^ test for non-continuous variables, according to distribution

*MMSE* Mini-Mental State Examination, *CIRS* Cumulative Index Rating Scale, *ADL* Activity of Daily Living, *IADL* Instrumental Activity of Daily Living, *GAS-10* Geriatric Anxiety Scale 10 items, *CL-FODS* Collett–Lester Fear of Death Scale, *GDS-5* Geriatric Depression Scale 5 items

Age was similar among patients recruited in Switzerland and in Italy, however, all the patients recruited in Italy were females, hospitalized because of hip fracture and had less comorbidity (CIRS 19.4 ± 6.3 in Switzerland and 9.2 ± 3.0 in Italy).

Among recruited patients, 17 (26.5%) chose to use phone calls and 47 (73.5%) video calls. Patients using phone and video calls did not differ significantly in their clinical characteristics except for the level of general anxiety that was higher in patients choosing video calls. Indeed, all four patients with GAS-10 scores above the cut-off suggests abnormal anxiety selected video calls (Table 1).

### Patients’ and relatives’ acceptance of video calls at baseline

**Fig. 1:**
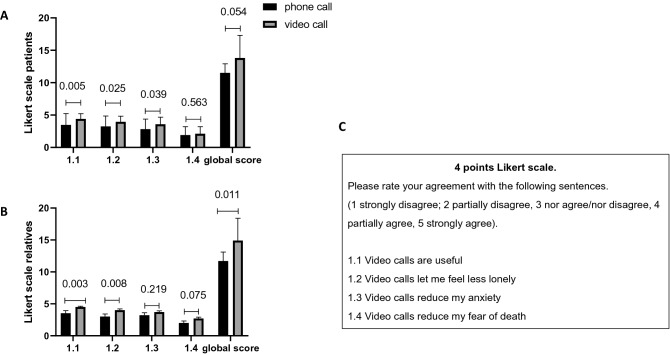
Acceptance of video and phone calls by patients and relatives at baseline. Answers to the 4-point Likert scale by patients (**A**) and their relatives (**B**); **C** shows the 4-point Likert scale affirmations. Bar represents mean values, SD is shown, and significant *p* values obtained by one-way ANOVA are shown

At baseline, patients’ acceptance of video calls differed between those who selected this mean of communication and those who preferred phone calls (Fig. 1A). Compared to patients who selected phone calls, those who selected video calls were more likely to consider them as useful, as making them feel less lonely and as reducing their anxiety.

Results from relatives’ acceptance of video calls comparing those who planned to use video vs phone calls showed differences across groups similar to those observed in patients with regards to usefulness and loneliness (Fig. 1B), except for the reduction of anxiety that remained not affected.

### Patients’ acceptance of video calls after the 1-week study period

The use of video calls during the study period increases the appreciation of their utility (*p* = 0.004) and of their role in reducing the feeling of loneliness (*p* = 0.009), whereas there was no significant difference in patients using phone calls. (Supplemental Table 1).

### Patients’ anxiety, fear of death, and mood outcome after the 1-week study period

**Fig. 2:**
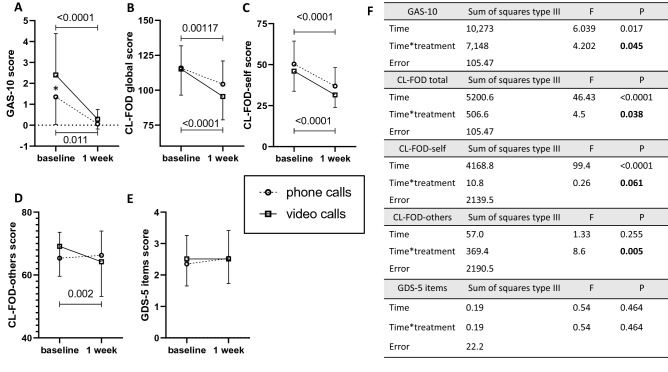
Effects of communication using video or phone calls on patients’ general anxiety, anxiety due to the fear of death and mood. Graphs showing mean and SD for anxiety (GAS-10, **A**), fear of death and dying global (CL-FOD-global, **B**), fear of self-death and dying (CL-FOD-self, **C**), fear of other’s death and dying (CL-FOD-others, **D**) mood (GDS-5, **E**). Significant differences between baseline and follow-up have been calculated by two-way ANOVA for a repeated measure, the difference between groups at baseline and follow-up has been calculated with Sidak’s multiple comparison test, p significant t values are indicated by *. **F** shows statistical results for the two-way ANOVA for repeated measures test

General anxiety was significantly reduced from baseline to the end of the study in both groups of participants using video and phone calls. However, the magnitude of this reduction was significantly more important in patients using video than phone calls (Fig. 2A,F). In addition, among the four patients from the video calls group who scored above the clinical threshold to define the presence of abnormal anxiety at baseline, one reduced his level of anxiety below this threshold.

The post-hoc power calculation for the GAS-10 scale with a significant alpha at 0.05 is 90%.

Fear of death significantly decreased over time in participants from both video and phone calls groups. The reduction appeared to be larger in participants from the video calls group for overall fear of death, as well as for fear of self-death, and fear of others’ death (Fig. 2B–D,F). Post-hoc analysis provided a statistical power of 62% for this outcome.

Finally, no significant effect was observed on mood (GDS-5) within as well as across groups (Fig. 2E); however, the statistical power for this outcome was very low (25%).

### Relatives’ acceptance of video calls after the 1-week study period

Relatives using video calls during the study increased their appreciation toward this communication technology; in particular, they found that there is a useful communication tool (*p* < 0.001) that is able to reduce their anxiety (*p* = 0.013) (Supplemental Table 2). Post-hoc power calculation for the 4-point Likert scale with a significant alpha at 0.05 is 89%.

### Relatives’ anxiety and fear of death

**Fig. 3:**
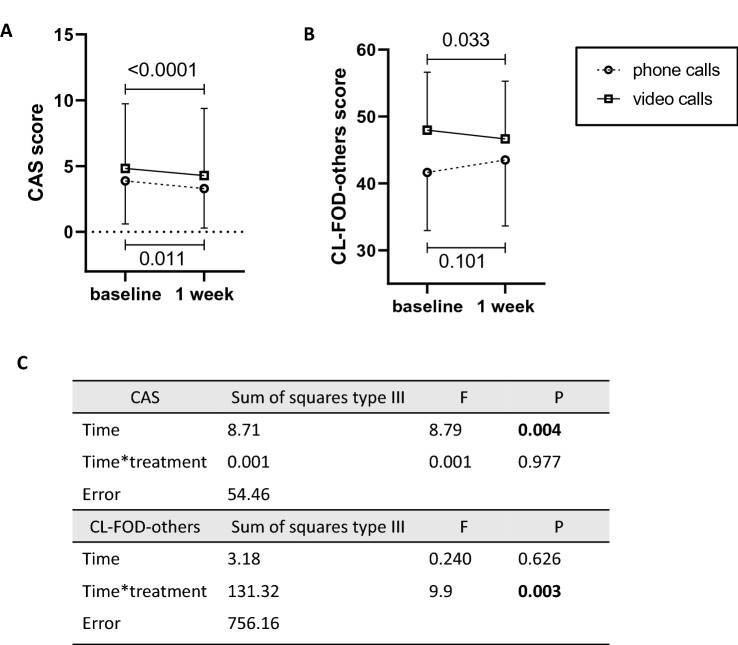
Effects of communication using video or phone calls on relatives’ general anxiety and anxiety due to the fear of death of others. Graphs showing mean and SD for anxiety (CAS, **A**), fear of other’s death and dying (CL-FOD-others, **B**). Significant differences between baseline and follow-up have been calculated by two-way ANOVA for the repeated measure. **C** Statistical results for the two-way ANOVA for repeated measures test

General anxiety, as measured by CAS, significantly decreased over time in both groups, without any difference across video and phone calls groups (Fig. 3A, C). None of the enrolled relatives showed clinically significant anxiety (CAS score ≥ 30). Post hoc analysis revealed a statistical power of only 24.6% for this outcome.

In contrast, fear of the loved ones’ death evolved in opposed directions in the two groups of relatives, with a significant beneficial effect among video vs phone calls users (Fig. 3B, C). Indeed, fear of death significantly decreased among relatives who used video calls whereas it increases among relatives who used phone calls. The post-hoc power calculation for this outcome with a significant alpha at 0.05 was 84%.

General anxiety and fear of death were weakly correlated in both patients and families. SpecificallyGAS-10 was significantly correlated with fear of the loved one’s death in patients (at baseline, *R* = 0.27, *p* = 0.031 and at follow-up, *R* = 0.30, *p* = 0.029). In families, CAS was significantly correlated with fear of the loved one’s death both at baseline (*R* = 0.49, *p* < 0.0001) and at follow-up (*R* = 0.55, *p* < 0.0001).

## Discussion

In hospitalized critically ill patients, interaction with relatives significantly affect the experience of illness and communication interventions reduce the development of stress-related symptoms in both patients and relatives [4]. Indeed, visit ban measures also affected patients’ relatives as well as healthcare professionals who all had to adopt new communication strategies. Among those, video calls were used to address patients’ and families’ distress. Compared to phone calls, video calls may allow a more natural and complete form of communication [9]. Patients and relatives using video calls may not only be able to speak and to hear each other but also to look at facial expressions, thus enriching the exchanges with non-verbal communication strategies [9].

SILVER investigates the acceptance of video calls as compared to phone calls to maintain communication during the ban of visits due to the COVID-19 pandemic and the effects of using video versus phone calls on anxiety, fear of death and mood in older hospitalized patients and their relatives.

The first contribution of SILVER is to show that, contrary to what has been reported in another previous study [12], the majority of these patients selected video calls rather than phone calls despite the burden of an acute condition, age and comorbidity. Sacco and colleagues [12] claim that the presence of a pre-existing experience with video calls may influence the patients’ choice. We do not have information on pre-existing experience with video call; hence, we cannot support or deny this hypothesis. However, our data show that the use of video calls increase the appreciation of this communication technology among patients and relatives.

This study also provides original information about the specific domains where video calls appear to be most beneficial for patients and for their relatives as compared to phone calls. The most striking result is certainly the significant benefit reported by both patients and relatives about their fear of the death of their loved one. Indeed, a unique contribution of this study is to show diverging trends over the study period among video (decreased fear) vs phone (stable or increased fear) call users. We hypothesize that seeing one's own loved one is more effective than just listening to him/her voice in reassuring about his/her state of health. An additional important contribution of SILVER is to highlight the link between the reported benefit of video calls on fear of the loved one’s death and the observed benefit on patients’ and relatives’ anxiety resulting from the visits ban. Although results from the GAS-10 showed decreased anxiety over time in both groups of patients, this improvement was more pronounced among those using video calls. Similarly, relatives who used video calls were also more likely to positively appreciate the role of video calls in reducing their anxiety than those using phone calls.

Overall, these results lend support to using video calls in showing added value to patients’ and their relatives’ fear of death and anxiety. Furthermore, these results strongly challenge the assumption that older patients are refractory to video calls as a communication tool with their relatives [12].

The main limitation of this study is its sample size; nevertheless, we achieved adequate statistical power for 4 out of six outcomes. Moreover, the exclusion of patients with moderate to severe form of dementia exclude a non-negligible part of patients hospitalized in the acute care geriatric unit. Considering the peculiarity in the communication and evaluation of patients living with dementia, a study planned ad hoc may be more adequate. A randomized and controlled approach would have been more appropriate to avoid selection bias, however, we found it ethically inacceptable to impose a type of communication to patients and their relatives. Furthermore, the comprehensive geriatric methodology is the gold standard approach to drive healthcare-related decisions in older adults and it accounts for patients’ goals and preferences to maximise participation [27]. The inclusion of three centers from two countries providing acute as well as post-acute care may be considered both as a limitation and as a strength of the study. We cannot exclude the influence of different cultures and different types of populations on our outcomes; however, it is reasonable to hypothesize that the influence is maintained over the whole study, so the culture may influence differences at baseline and not the effect of the longitudinal intervention. Age and gender differences between patients enrolled in Italy and in Switzerland are explained by the type of center included in Italy, in the orthogeriatric unit femoral fractures are the cause of admission and it is more frequent in females because of the higher incidence of osteoporosis in this population. The inclusion of two countries may also be considered a strength of our study as it allows a generalization of our findings. The inclusion of different countries also provides insight into acceptance across different cultures, even though the limited sample size precluded investigating potential differences across countries. The study also was performed in a real-world situation with simple standardized procedures, validated and internationally recognized scales to quantify primary and secondary outcomes that would facilitate its replication in other health care environments.

## Conclusions

Results of the SILVER study show that old adults and their relatives accept and appreciate the use of video calls as a communication tool during hospitalization; moreover, SILVER suggests that video calls may be useful in relieving general and fear of death anxiety in old hospitalized patients. Similarly, SILVER suggests that the use of video calls may reduce the anxiety due to the fear of their loved one’s death in relatives.

Thus implementing video calls as a communication tool in hospitals may be useful for old adults even without visit restrictions to keep social relationships with relatives unable to come to the hospital to visit their relatives.

## Supplementary Information

Below is the link to the electronic supplementary material.

Supplementary file1 (DOCX 18 KB)

## Data Availability

The data that support the findings of this study are available from the corresponding author, [PD], upon reasonable request.
