# Supplementary material for: Fighting social isolation in times of pandemic COVID-19: the role of video calls for older hospitalized patients
Source: Aging Clin Exp Res. 2022 Jul 6;34(9):2245–53. doi: 10.1007/s40520-022-02188-8 (PMC9261146; doi:10.1007/s40520-022-02188-8)
Supplement: Supplementary file 1 — Supplementary file1 (DOCX 18 KB) [file 40520_2022_2188_MOESM1_ESM.docx]

**Suplemental table 1.** **Patients’ acceptance of video-calls, from the 4-point Likert scale**.
The table shows multiple T-test and Two-way ANOVA for multiple measure results. Values are shown as mean ± SE and 95% CI of the difference between basal and 1 week of study.

* Denotes differences between baseline and 1 week; Significant values are in bold.

|  | **Video-calls are useful** | | | | | |
| --- | --- | --- | --- | --- | --- | --- |
|  | **Phone-calls** | | **Video-calls** | | **Two-way ANOVA** | |
|  | *Mean ±*  *SE* | *95%*  *CI of difference* | *Mean ± SE* | *95% CI*  *of difference* | *Effect of* | *p* |
| **Baseline** | 3.5±1.7 | -1.497 to 1.967 | **4.4±0.8** | **-0.498 to 0.080*** | **Time** | 0.759 |
| **1 week** | 3.1±1.9 |  | **4.7±0.7** |  | **Interaction** | **0.004** |
|  | **Video-calls let me feel less lonely** | | | | | |
|  | **Phone-calls** | | **Video-calls** | | **Two-way ANOVA** | |
|  | *Mean ±*  *SE* | *95% CI*  *of difference* | *Mean ± SE* | *95% CI*  *of difference* | *Effect of* | *p* |
| **Baseline** | 3.2±1.6 | -1.448 to 1.801 | 4.0±0.9 | -1.091 to 0.202 | **Time** | 0.251 |
| **1 week** | 3.1±1.9 |  | 4.4±0.8 |  | **Interaction** | **0.009** |
|  | **Video-calls reduce my anxiety** | | | | | |
|  | **Phone-calls** | | **Video-calls** | | **Two-way ANOVA** | |
|  | *Mean ±*  *SE* | *95% CI*  *of difference* | *Mean ± SE* | *95% CI*  *of difference* | *Effect of* | *p* |
| **Baseline** | 2.8±1.6 | -1.838 to 1.368 | **3.6±1.1** | **1.087 to 0.206*** | **Time** | **0.024** |
| **1 week** | 3.1±1.9 |  | **4.0±0.8** |  | **Interaction** | 0.251 |
|  | **Video-calls reduce my fear of death** | | | | | |
|  | **Phone-calls** | | **Video-calls** | | **Two-way ANOVA** | |
|  | *Mean ±*  *SE* | *95% CI*  *of difference* | *Mean ± SE* | *95% CI*  *of difference* | *Effect of* | *p* |
| **Baseline** | 1.9±1.3 | -0.8552 to 1.208 | 2.1±1.1 | -0.461 to 0.832 | **Time** | 0.251 |
| **1 week** | 1.8±0.8 |  | 1.9±0.8 |  | **Interaction** | 0.997 |
|  | **Likert 4 items global score** | | | | | |
|  | **Phone-calls** | | **Video-calls** | | **Two-way ANOVA** | |
|  | *Mean ±*  *SE* | *95% CI*  *of difference* | *Mean ± SE* | *95% CI*  *of difference* | *Effect of* | *p* |
| **Baseline** | 11.5±5.6 | -5.242 to 5.948 | **13.8±3.5** | **-1.272 to -0.004** | **Time** | 0.745 |
| **1 week** | 11.5±6.3 |  | **14.4±3.9** |  | **Interaction** | 0.260 |

**Supplemental table 2.** **Relatives’ appreciation of video-calls, from the 4-point Likert scale.**The table shows multiple T-test and Two-way ANOVA for multiple measure results. Values are shown as mean ± SE and 95% CI of the difference between baseline and the end of the 1-week study period.

* Denotes differences between baseline and the end of the 1-week study period; Significant values are in bold.

|  | **Video-calls are useful** | | | | | |
| --- | --- | --- | --- | --- | --- | --- |
|  | **Phone-calls** | | **Video-calls** | | **Two-way ANOVA** | |
|  | *Mean ± SE* | *95% CI*  *of difference* | *Mean ±*  *SE* | *95% CI*  *of difference* | *Effect of* | *p* |
| **Baseline** | 3.5±1.6 | -0.661 to 1.837 | 4.5±0.9 | -0.651 to 0.260 | **Time** | **0.033** |
| **1 week** | 2.9±1.9 |  | 4.7±0.7 |  | **Interaction** | **<0.001** |
|  | **Video-calls let me feel less lonely** | | | | | |
|  | **Phone-calls** | | **Video-calls** | | **Two-way ANOVA** | |
|  | *Mean ± SE* | *95% CI*  *of difference* | *Mean ±*  *SE* | *95% CI*  *of difference* | *Effect of* | *p* |
| **Baseline** | 2.8±1.8 | -1.072 to 1.425 | 4.5±1 | -0.866 to 0.0461 | **Time** | 0.502 |
| **1 week** | 3.0±1.7 |  | 4.1±1.2 |  | **Interaction** | 0.087 |
|  | **Video-calls reduce my anxiety** | | | | | |
|  | **Phone-calls** | | **Video-calls** | | **Two-way ANOVA** | |
|  | *Mean ± SE* | *95% CI*  *of difference* | *Mean ± SE* | *95% CI*  *of difference* | *Effect of* | *P* |
| **Baseline** | 3.2±1.6 | -0.719 to 1.778 | 3.6±1.3 | -0.630 to 0.281 | **Time** | 0.209 |
| **1 week** | 2.7±1.7 |  | 3.8±1.2 |  | **Interaction** | **0.013** |
|  | **Video-calls reduce my fear of death** | | | | | |
|  | **Phone-calls** | | **Video-calls** | | **Two-way ANOVA** | |
|  | *Mean ± SE* | *95% CI*  *of difference* | *Mean ± SE* | *95% CI*  *of difference* | *Effect of* | *P* |
| **Baseline** | 2±1.4 | -1.543 to 0.955 | 2.7±1.3 | -0.667 to 0.245 | **Time** | 0.115 |
| **1 week** | 2.3±1.8 |  | 2.9±1.3 |  | **Interaction** | 0.762 |
|  | **Likert 4 items global score** | | | | | |
|  | **Phone-calls** | | **Video-calls** | | **Two-way ANOVA** | |
|  | *Mean ± SE* | *95% CI*  *of difference* | *Mean ± SE* | *95% CI*  *of difference* | *Effect of* | *P* |
| **baseline** | 11.7±5.8 | -0.249 to 2.249 | **14.9±3.6** | **-1.446 to -0.535*** | **Time** | 0.975 |
| **1 week** | 10.7±6.8 |  | **15.9±3.3** |  | **Interaction** | 0.762 |
